# Threaded biliary inside stents are a safe and effective therapeutic option in cases of malignant hilar obstruction

**DOI:** 10.1186/1471-230X-13-31

**Published:** 2013-02-14

**Authors:** Osamu Inatomi, Shigeki Bamba, Makoto Shioya, Yosuke Mochizuki, Hiromitsu Ban, Tomoyuki Tsujikawa, Yasuharu Saito, Akira Andoh, Yoshihide Fujiyama

**Affiliations:** 1 Division of Gastroenterology, Shiga University of Medical Science, Seta-Tsukinowa cho, Otsu, Shiga, Japan; 2 Division of Endoscopy, Shiga University of Medical Science, Otsu, Shiga, Japan; 3 Division of Mucosal Immunology, Graduate School of Medicine, Shiga University of Medical Science, Otsu, Shiga, Japan

## Abstract

**Background:**

Although endoscopic biliary stents have been accepted as part of palliative therapy for cases of malignant hilar obstruction, the optimal endoscopic management regime remains controversial. In this study, we evaluated the safety and efficacy of placing a threaded stent above the sphincter of Oddi (threaded inside plastic stents, threaded PS) and compared the results with those of other stent types.

**Methods:**

Patients with malignant hilar obstruction, including those requiring biliary drainage for stent occlusion, were selected. Patients received either one of the following endoscopic indwelling stents: threaded PS, conventional plastic stents (conventional PS), or metallic stents (MS). Duration of stent patency and the incident of complication were compared in these patients.

**Results:**

Forty-two patients underwent placement of endoscopic indwelling stents (threaded PS = 12, conventional PS = 17, MS = 13). The median duration of threaded PS patency was significantly longer than that of conventional PS patency (142 vs. 32 days; *P* = 0.04, logrank test). The median duration of threaded PS and MS patency was not significantly different (142 vs. 150 days, *P* = 0.83). Stent migration did not occur in any group. Among patients who underwent threaded PS placement as a salvage therapy after MS obstruction due to tumor ingrowth, the median duration of MS patency was significantly shorter than that of threaded PS patency (123 vs. 240 days).

**Conclusions:**

Threaded PS are safe and effective in cases of malignant hilar obstruction; moreover, it is a suitable therapeutic option not only for initial drainage but also for salvage therapy.

## Background

Unresectable hilar cholangiocarcinoma has a very poor prognosis, with an overall survival of less than 6 months [1,2]. Patients with malignant hilar biliary strictures often present with obstructive jaundice, consequently requiring biliary drainage. Endoscopic indwelling stents have been widely used for the treatment of malignant hilar obstructions [3-5]. Plastic stents (PS) are more economical and easier to deliver and remove than uncovered metallic stents (MS). However, the patency duration of PS is generally shorter than that of uncovered MS because of food impaction and ascending bacterial infection from the duodenum [6-8].

Recently, various techniques have been reported for stent insertion and stent refinement for the prolongation of PS patency [9-14]. Placement of PS above the sphincter of Oddi (inside stents) was reportedly superior to conventional PS with respect to stent patency in cases of malignant bile duct obstruction [15]. However, another study showed that placement of the inside stent was associated with high rates of stent migration and occlusion [16]. Therefore, the ideal choice of stent for use in cases of malignant hilar obstruction remains controversial.

To overcome the disadvantages of PS and to facilitate delivery and removal of the so-called inside stents, we first attach a nylon thread to a conventional PS and then attempt the placement of this threaded stent above the sphincter of Oddi (threaded inside plastic stents, threaded PS). In this study, we retrospectively evaluated and compared the safety and efficacy of threaded PS placement with those of conventional MS and PS placement in cases of malignant hilar obstruction.

## Methods

### Patients

Between April 2007 and March 2011, 42 cases with unresectable malignant hilar obstruction underwent endoscopic indwelling stent placement, including threaded PS (n = 12), conventional PS (n = 17), and MS (n = 13) placement. Diagnosis was based on CT scans, MRCP, and endoscopic retrograde cholangiography. Malignancy was histologically confirmed in all patients. Hilar biliary strictures were classified according to the Bismuth classification [17]: type I, obstruction at the bifurcation but with communication between the left and right hepatic ducts; type II, separate obstruction of the left and right main hepatic ducts; type III, obstruction extending to at least the secondary branches of either the right or left hepatic duct, with the contralateral ductal system remaining intact; and type IV, obstruction involving the bilateral secondary branches or multifocal strictures.

All patients were considered to have unresectable malignancies after the evaluation of tumor extent, distinct metastasis, and advanced age. Standard gemcitabine or S-1-based chemotherapy was administered after successful biliary drainage in patients with a good performance status. No patient received radiation therapy. Follow-up continued until patient death or study completion.

All patients provided informed written consent prior to undergoing stent placement. The study was conducted in agreement with the Declaration of Helsinki and received approval (No. 21–91) from the ethics committee of Shiga University of Medical Sciences and conformed to its guidelines.

### Endoscopic procedure for stent insertion

All endoscopic procedures were performed by two experienced endoscopists. The patients were moderately sedated (intravenous midazolam and pentazocine). For all biliary stent placement techniques, JF260V or TJF-240 duodenoscopes (Olympus, Tokyo, Japan) were used. Basically, we firstly underwent endoscopic nasobilliary drainage for adequate drainage and accurate diagnosis, and then, after a few days, inserted a suitable stent for a particular stenosis. The type of stent that inserted initially is MS or conventional PS in the early period of this study (from April 2007 to September 2009), and threaded PS in the late (from October 2009 to March 2011).

The stents were inserted over a guidewire after endoscopic retrograde cholangiography. The length of the stent was selected so that either end of the stent was approximately 1 cm beyond the tumor margin. Endoscopic sphincterotomy was performed additionally in patients who received uncovered MS. Multiple stents were placed in cases suffering from high-grade hilar biliary strictures. Multiple MS were placed by a partial stent-in-stent technique. For a unilateral stent placement, the side for the proximal end of the stent was judged by the surgeon’s discretion according to the information on lobar volume and angle.

The procedures used for the insertion of each stent are described below.

1) Threaded PS placement

**Figure 1:**
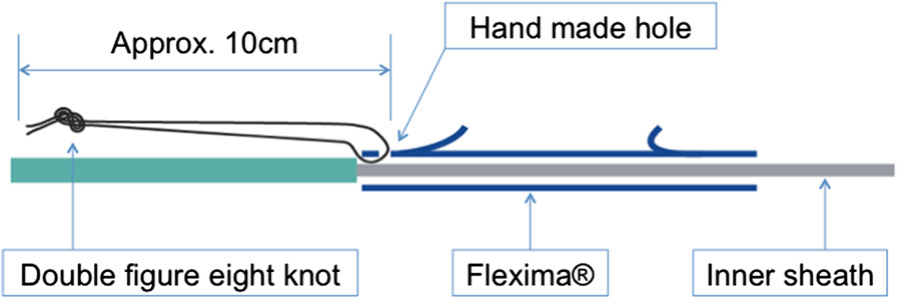
**The design of threaded inside plastic stent (Threaded PS).** A nylon monofilament thread (size 3/0) is attached to the conventional PS (Flexima, Boston Scientific) by using a puncher.

**Figure 2:**
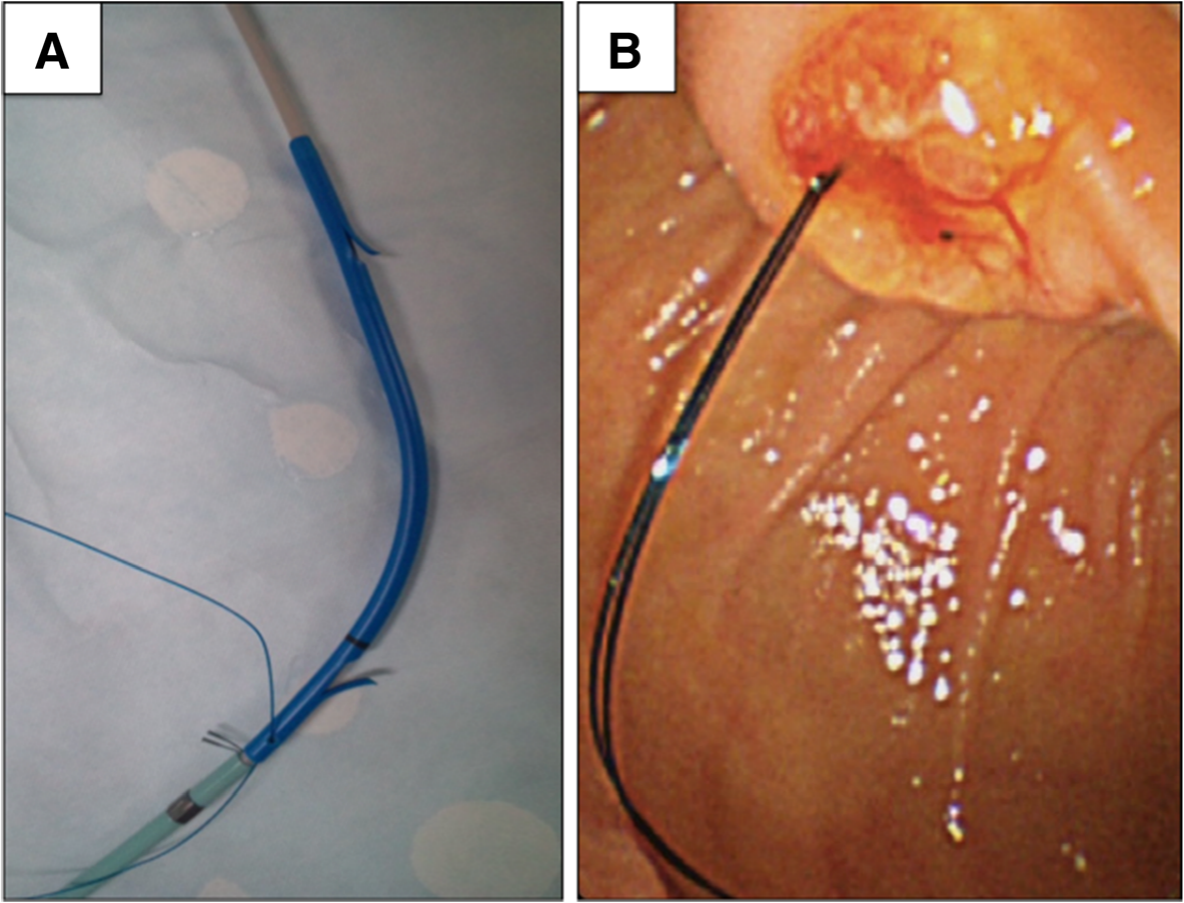
**Picture of threaded inside plastic stent (Threaded PS). A**. The threaded inside stent before insertion **B**. Endoscopic view of the thread, which is placed outside the papilla.

A double figure eight knot was used to attach a nylon monofilament thread (size 3–0, 10 cm long) to a conventional PS (7Fr, 5–10 cm, Flexima, Boston Scientific, Natick, MA, USA) through a hole made by a puncher (Figure 1). This threaded stent was inserted by the standard procedure using a guidewire and pusher catheter, and then placed above the sphincter of Oddi with the attached nylon thread remaining outside the papilla. This enabled the surgeon to exchange stents easily by grabbing the protruding thread (Figure 2).

2) Conventional PS

Double pigtail stents (7Fr or 8Fr, 5–10 cm, Cook Medical Inc., Bloomington, IN, USA) or Flexima stents (7Fr, 8.5Fr, or 10Fr, 5–10 cm, Boston Scientific) were selected at the surgeon’s discretion. This stent was inserted by the similar procedure as threaded PS, and then placed across the sphincter of Oddi with the distal end of the stent protruding into the duodenum.

3) MS

Uncovered MS (ZEOSTENT plus or JOSTENT SelfX, 4–8 cm; Zeon Medical Inc., Tokyo, Japan) were selected at the surgeon’s discretion**.** The stent was inserted using the delivery system, and then placed above the sphincter of Oddi after the delivery catheter was released.

### Outcome measurement

For assessment of clinical response, successful cases of biliary decompression were defined as those in which serum total bilirubin declined to a normal level (<1.2 mg/dL) or less than half of the level before stent placement. Stent occlusion was essentially diagnosed by endoscopic retrograde cholangiography when a patient developed cholangitis or jaundice after stent placement.

### Efficacy of threaded PS placement as a salvage therapy in cases of MS obstruction

Three patients who initially received MS underwent threaded PS placement because of stent obstruction caused by tumor ingrowth. On the other hand, one patient who initially received threaded PS underwent MS placement because of stent obstruction caused by tumor hemorrhage. To evaluate the efficacy of threaded PS placement as a salvage therapy in cases of MS obstruction, the duration of stent patency was compared among these patients.

### Statistical analysis

Student’s t-test was used to compare baseline variables between each group. The median duration of stent patency was analyzed by the Kaplan–Meier method, and the results were compared by the logrank test. A *P*-value of <0.05 was considered statistically significant. All statistical analyses were performed with GraphPad PRISM version 4.03 software (MDF Co., Ltd., Tokyo, Japan).

## Results

### Patient characteristics

Patient characteristics are presented in Table 1. There were no significant differences in age, sex, cause of biliary obstruction, Bismuth classification, and the presence or absence of chemotherapy among the three groups. Multiple stent placement was attempted only in the threaded PS and MS groups, and there was no significant difference in the number of patients (threaded PS: n = 2, MS: n = 4).

**Table 1:** Patient characteristics

	**Threaded PS (N = 12)**	**PS (N = 17)**	**MS (N = 13)**
Sex			
Male	7	8	7
Female	5	9	6
Age (years) mean ± SD	67.5 ± 11.7	66.1 ± 12.2	69.2 ± 13.2
Diagnosis			
Bile duct cancer	5	6	7
Gall bladder cancer	4	8	4
Others	3	3	2
Bismuth classification			
Type I	4	6	4
Type II	1	1	3
Type III	2	2	2
Type IV	5	8	4
Serum total bilirubin (mg/dl) mean ± SD	7.5 ± 5.6	6.2 ± 2.9	5.4 ± 3.2
Chemotherapy received *	5 (50%)	11 (65%)	4 (31%)
Gemcitabine	4	7	2
S-1	1	7	4

* Chemotherapy include combined therapy of gemcitabine and S-1.

### Overall stent patency

**Figure 3:**
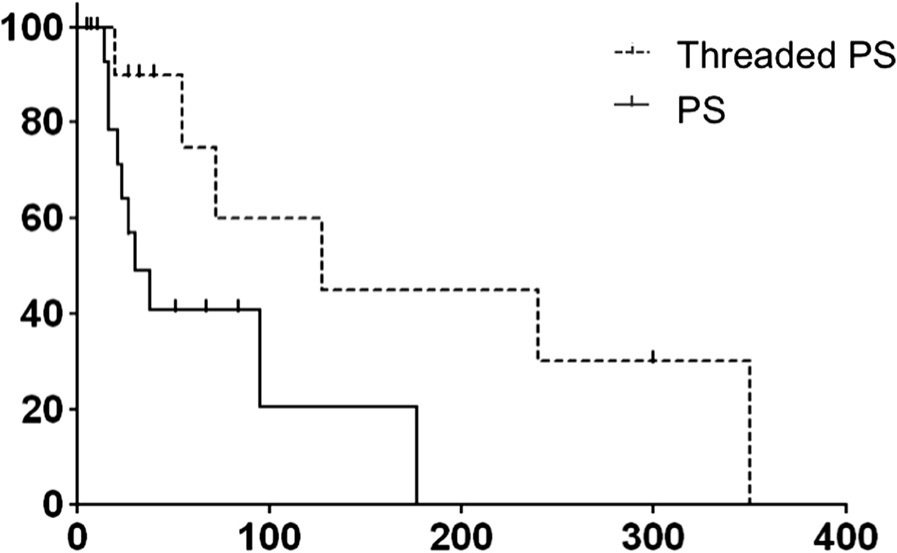
**Cumulative patency durations of the plastic stents (PS) and the threaded inside plastic stents (Threaded PS).** Kaplan–Meier estimation of the cumulative durations of stent patency in the conventional PS (solid line; median duration, 32 days) and threaded PS groups (dotted line; median duration, 142 days).

**Figure 4:**
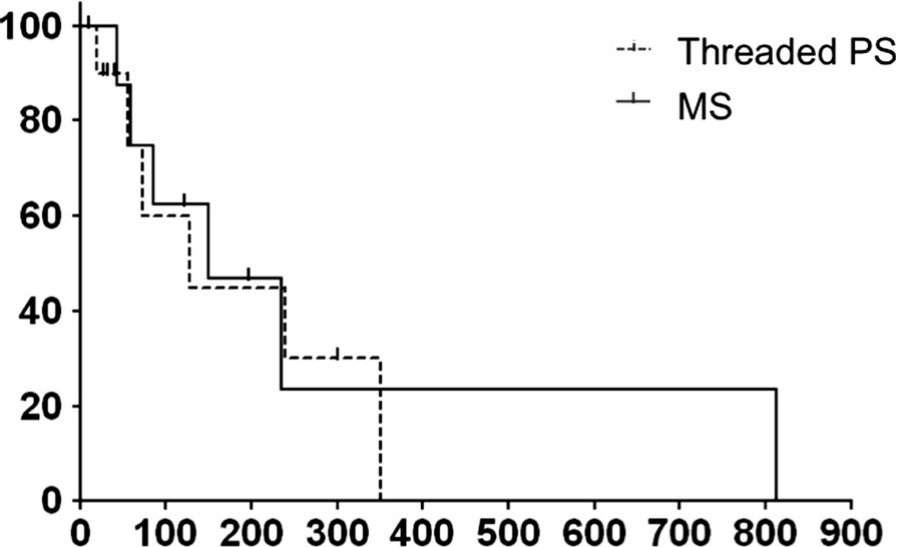
**Cumulative patency durations of the metallic stents (MS) and the threaded inside plastic stents (Threaded PS).** Kaplan–Meier estimation of the cumulative durations of stent patency in the MS (solid line; median duration, 150 days) and threaded PS groups (dotted line; median duration, 142 days).

All procedures for stent insertion were successful in each group. The median duration of stent patency was significantly longer in the threaded PS group than in the conventional PS group (142 days vs. 32 days; *P* < 0.05) (Figure 3). On the other hand, the median duration of stent patency in the threaded PS group was similar to that in the MS group (142 days vs. 150 days; *P* = 0.83) (Figure 4). Multiple stent placement and endoscopic sphincterotomy were not significantly related to median duration of stent patency in the threaded PS and MS group patients (164 days with multiple stent placement vs. 140 days without, *P* = 0.60; 153 days with endoscopic sphincterotomy vs. 125 days without, *P* = 0.52).

### Salvage therapy

Three patients who had undergone MS placement received threaded PS because of stent obstructions caused by tumor ingrowths (Patient 1, 2, 4 in Table 2). The overview images and durations of threaded PS and MS patency in these patients, who underwent threaded PS placement as a salvage therapy, are shown in Table 3. The mean duration of MS patency (first intervention) was shorter than that of threaded PS patency in these patients (123 vs. 240 days). On the other hand, one patient underwent MS placement after threaded PS obstruction caused by tumor hemorrhages; of these, the duration of MS patency was 124 days, whereas those of threaded PS patency was 6 days (Patient 7 in Table 2).

**Table 2:** Details of patients with threaded PS placement

	**Primary lesion**	**Performance status**	**Stage**	**Bismuth classification**	**Initial stent**	**Multiple stent**	EST*	**Chemotherapy**	**Patency period**	**Outcome**
**1**	**Bile duct**	**2**	**IV**	**IV**	**N**	**N**	**Y**	**No**	**350**	**Death**
**2**	**Bile duct**	**2**	**IV**	**IV**	**N**	**Y**	**Y**	**No**	**300**	**Death**
**3**	**Gall bladder**	**0**	**IV**	**I**	**N**	**N**	**N**	**GEM**	**270**	**Obstruction**
**4**	**Bile duct**	**0**	**IV**	**IV**	**N**	**Y**	**Y**	**GEM**	**72**	**Obstruction**
**5**	**Bile duct**	**1**	**IV**	**IV**	**N**	**N**	**Y**	**No**	**108**	**Death**
**6**	**Pancreas**	**1**	**IV**	**I**	**Y**	**N**	**N**	**GEM**	**56**	**Obstruction**
**7**	**Gall bladder**	**3**	**IV**	**II**	**Y**	**N**	**N**	**No**	**6**	**Obstruction by Coagulation**
**8**	**Gall bladder**	**1**	**IV**	**III**	**N**	**N**	**N**	**S-1**	**127**	**Obstruction**
**9**	**Bile duct**	**2**	**III**	**III**	**Y**	**N**	**N**	**GEM**	**41**	**Death**
**10**	**Pancreas**	**2**	**IV**	**I**	**Y**	**N**	**N**	**No**	**32**	**Death**
**11**	**Gall bladder**	**1**	**IV**	**IV**	**N**	**N**	**N**	**No**	**80**	**Obstruction**
**12**	**Pancreas**	**2**	**III**	**I**	**Y**	**N**	**N**	**No**	**290**	**Alive**

EST, endoscopic sphincterotomy; GEM, gemcitabine.

* These cases have already received endoscopic sphincterotomy in the earlier stent placement of MS or PS before threaded PS insertion.

**Table 3:** The durations of stent patency for three cases underwent threaded PS placement after MS occlusion

	**MS patency (Days)**	**Post-salvage patency (Days)**
Case 1	85	350
Case 2	234	300
Case 3	59	72

### Complications

Threaded PS placement in an appropriate position between the stenosis and sphincter of Oddi was technically easy because the position was adjusted by grasping the attached thread with a forceps after the stent was released from the scope. There were no threaded PS migrations even in cases that received multiple stents.

Major complication such as perforation or bleeding did not occur in cases that received endoscopic sphincterotomy.

## Discussion

The advantages of this custom-made stent include uncomplicated positioning, which is not across the sphincter of Oddi, and ease of fabrication and removal, which is facilitated by grabbing the attached thread. In addition, threaded PS placement and exchange can be performed without endoscopic sphincterotomy, which increases the risk of complications like bleeding, perforation, pancreatitis, or ascending infection [18-20]. In the present study, we showed that the median duration of threaded PS patency was longer than that of conventional PS patency, and that there was no significant difference between the duration of threaded PS and MS patency.

Although conventional PS is still widely used for endoscopic biliary drainage, it sometimes requires frequent stent exchanges at intervals of 1–4 months because of stent obstruction with biliary sludge [21,22]. The main reasons for PS obstruction seem to be the smaller stent diameter and the bacterial biofilm created by ascending infection from the duodenum, leading to biliary sludge formation [7,18]. Given that the threaded PS diameter in our study was only 7 Fr, its placement above the intact sphincter of Oddi presumably avoided this duodenobiliary reflux, leading to longer patency.

The efficacy of inside stents (without an attached thread) in cases of malignant bile duct obstruction has been investigated in other studies. In a randomized clinical trial, Pedersen et al*.* reported that the median patency duration of stents placed above the sphincter of Oddi was comparable to that of stents placed across the sphincter of Oddi [16]. However, the migration rate of stents placed above the sphincter of Oddi was 58% in their study. On the other hand, Uchida et al. reported that the median patency duration of stents placed above the sphincter of Oddi was superior to that of stents placed across the sphincter of Oddi (255 vs. 82 days), and the respective occlusion rates were 37.5% and 93.8% [15]. Although their results with respect to stent patency were satisfactory, endoscopic sphincterotomy was required for stent exchange in most cases of stent occlusion.

In our study, four cases in the threaded PS group had received initial treatment with MS, PS or endoscopic nasal drainage with endoscopic sphincterotomy. Although stent patency was not significantly different between patients who do or do not undergo endoscopic sphincterotomy in all cases that underwent threaded PS placement, the rate of complications, such as bleeding, perforation and pancreatitis, is reportedly 1%–10% [19,23,24]. Therefore, this option should be avoided wherever possible.

MS with larger diameters have been developed during the past two decades [25,26], and these have proven to possess longer durations of patency than those of conventional PS [13,27,28]. In a randomized controlled study, Wagner et al. reported superior performance of uncovered MS in cases of malignant hilar obstruction; furthermore, they found that the occlusion rate of uncovered MS was lower than that of PS (18.9% vs. 50%) [3]. However, clinical problems with MS placement often occur with respect to cost, stent occlusion by tumor ingrowth or overgrowth and mucosal hyperplasia induced by chronic inflammation [29,30]. Additionally, endoscopic sphincterotomy is necessary for MS placement in most cases. In the present study, the duration of threaded PS patency was shown to be superior to that of PS patency and noninferior to that of MS patency.

In patients who receive MS, another problem is reintervention in cases of stent occlusion. Uncovered MS cannot be removed and are difficult to insert as a second mode of drainage using the stent-in-stent technique, because the first stent is often deployed within the bile duct lumen [31]. In the present study, we also evaluated the efficacy of threaded PS placement used as a second mode of drainage in cases of MS obstruction. Our results showed that the duration of threaded PS patency was appreciably longer than that of MS patency in all patients. The cause of occlusion in these patients was rapid tumor ingrowth. In contrast, threaded PS quickly occluded because of coagulation caused by tumor hemorrhage in one patient; he received MS as a second mode of drainage. These results suggest that threaded PS are probably superior to MS only for tumors with certain biological characteristics. Moreover, overall survival of these 3 patients was an average of 280 days despite tumor progression. Therefore, we recommend threaded PS placement in patients with aggressive tumor that progress locally in the biliary duct without distant metastasis.

The present analysis was retrospective; therefore, selection bias may have affected the outcome, although all cases in this study were consecutive and the same endoscopists made all decisions regarding stent type during the data collection period. In addition, the study sample was small. Further studies, such as randomized controlled studies with a larger study sample, are warranted.

Although surgical resection is the only curative option for hilar cholangiocarcinoma, 5-year survival following resection reportedly ranges from 20% to 40% [32]. Most patients need palliative therapy such as endoscopic drainage to prolong survival with a good quality of life [26,33].

Therefore, endoscopists should select the type of drainage on the basis of a patient’s performance status, their expected survival, the technical difficulty, and the characteristics of stent occlusions.

## Conclusions

In conclusion, our data showed that threaded PS placement was safe and effective in cases of malignant hilar obstruction. It can be a suitable therapeutic option not only for initial drainage but also for salvage therapy.

## Competing interests

The authors declare that they have no competing interests in relation to this manuscript.

## Authors’ contributions

OI was involved in analyzing the results and writing the manuscript. SB and MS participated in the design of the study and performed the statistical analysis. YM and HB helped procedure for ERCP. YS and TT advised technical problem for delivering the stents as well as revising the manuscript. AA and YF conceived of the study, and participated in its design and coordination and helped to draft the manuscript. All authors read and approved the final manuscript.

## Pre-publication history

The pre-publication history for this paper can be accessed here:

http://www.biomedcentral.com/1471-230X/13/31/prepub
